# Comparison of Surface Morphology and Topography of Additively Manufactured SS 316L Steel After AWJM in Dependence on Layer Orientation

**DOI:** 10.3390/ma19061255

**Published:** 2026-03-22

**Authors:** Radoslav Vandžura, Matúš Geľatko, Marek Čornanič, Vladimír Simkulet, František Botko

**Affiliations:** Faculty of Manufacturing Technologies, Technical University of Košice, 080 01 Prešov, Slovakia; matus.gelatko@tuke.sk (M.G.); marek.cornanic.2@student.tuke.sk (M.Č.); vladimir.simkulet@tuke.sk (V.S.); frantisek.botko@tuke.sk (F.B.)

**Keywords:** abrasive waterjet, additive manufacturing, stainless steel, topography, morphology

## Abstract

Additively manufactured stainless steels are gaining considerable attention in the production of complex components, especially in the aerospace, food production, energy, and biomedical industries. Machining and achieving the desired surface properties of such materials remains a challenge. Abrasive waterjet machining technology appears to be one of the options due to the advantages it brings. Removing support structures and separating individual parts is also one of the possible applications of this technology. This study investigates the effects of process parameters for individual cut qualities (Q1–Q5) of abrasive waterjet on the surface properties of additively manufactured stainless steel (SS 316L) specimens, considering the different mechanical properties of the material due to the direction of layering of the material during its production. Experimental specimens were prepared by selective laser melting technology with parameters ensuring the best possible quality of the resulting part. The results of the study showed changes in the topography of the machined surface, especially in the roughness parameters. Scanning Electron Microscopy and Energy Dispersive X-ray Spectroscopy analysis proved the presence of fragmented abrasive particles in the cut areas.

## 1. Introduction

Abrasive waterjet machining (AWJM) is a high-energy, unconventional cutting process that utilizes a high velocity water jet to accelerate abrasive particles which remove the material. Compared to conventional cutting processes, AWJ offers several advantages: negligible heat-affected zone, low cutting forces, and the ability to cut very hard or highly reflective materials and create narrow cuts with complex contours [1,2,3]. Janković et al. pointed out that AWJ can achieve a high cut quality on a wide range of engineering materials, with surface properties largely controllable by the appropriate choice of process parameters and nozzle configuration [4]. The final cut quality is understood as a combination of cut geometry and surface integrity. In AWJ, it is primarily controlled by water pressure, abrasive mass flow, traverse speed, stand-off distance, and nozzle diameters [4,5]. In austenitic stainless steels, AWJ is widely used for contour cutting and the production of near-net-shape products. Llanto et al. systematically analyzed the cutting of AISI 304L and showed that waterjet pressure, abrasive mass flow, traverse speed, and material thickness have a statistically significant effect on surface roughness and material removal rate; material thickness was found to be the dominant factor for both responses [5]. Löschner et al. investigated the cutting of 10 mm thick AISI 316L steel and confirmed that increasing the traverse speed leads to a significant deterioration of the surface roughness, especially in the lower cut zone, while decreasing the traverse speed reduces the jet energy loss and improves the surface quality [6]. Parthiban et al. optimized the AWJ cutting of 2 mm AISI 316L material and showed that the abrasive flow rate, cutting speed and stand-off distance significantly affect the upper and lower cut widths and edge quality, and developed predictive models of the cut geometry based on these parameters [7]. Further experimental studies of AWJ confirm that the traverse speed, abrasive mass flow rate and stand-off distance are among the key factors determining the surface roughness of steels and other structural materials [8]. The cut quality is often expressed in terms of roughness parameters (e.g., Ra, Rz) and the visual appearance of individual cut areas. A typical feature of an AWJ cut is the existence of a relatively smooth upper zone and a progressively more sheared lower zone, as the jet loses kinetic energy with increasing depth [4]. In industrial practice, many AWJ systems classify the quality of the cut surface using quality classes Q1–Q5, where Q5 represents the highest, surface quality (smooth) and Q1 a rough, fast cut, in accordance with standards [9]. In the CAM software (iGEMS 2021; Borås, Sweden) of AWJ machines, Q-levels are usually implemented through various combinations of traverse speed and other technological parameters. In parallel with the development of AWJ, additive manufacturing, especially laser powder bed fusion (L-PBF) of metal alloys, has established itself as a promising technology to produce geometrically complicated parts. However, additively manufactured components are prone to process defects, such as high initial surface roughness, porosity, residual stresses, and anisotropy of mechanical properties [10]. The orientation of the printing direction, scanning strategy, beam energy and layer thickness significantly affect the microstructure, morphology, and defect density, which in turn determines the mechanical behavior and machinability of the alloy [10]. The layered nature of the process leads to a pronounced “staircase” effect and the presence of partially melted particles on the side surfaces, which are unacceptable from the point of view of functional requirements in many applications and require post-processing operations for surface finishing, for example in biomedical or food industry components [11]. Mechanical and abrasive-based post-processing methods therefore play a key role in qualifying AM metal parts for real-world use. A recent review by Medibew et al. comprehensively mapped conventional and unconventional abrasive machining and finishing techniques applied to additively manufactured metal parts, including grinding, milling, polishing, lapping, honing, sandblasting, abrasive jet machining, magnetic abrasive finishing, and electrochemical machining [12]. The authors have shown that these processes can reduce surface roughness, with milling and grinding often achieving roughness reductions of more than 90% for LPBF 316L, Ti-6Al-4V, and Inconel 718 [12]. For 316L stainless steel alloy, Aziz et al. have comprehensively reviewed strategies for optimizing AM process parameters, post-processing treatments to reduce defects [10]. They emphasized that the SLM (Selective Laser melting) process can achieve fine microstructure and high dimensional accuracy, and is particularly sensitive to residual stresses, porosity, and surface roughness. Some of the negative aspects can be reduced by heat treatment, and the combined effect of print orientation and selected post-processing procedure on surface integrity and fatigue properties of 316/316L is not yet fully understood. The material removal mechanisms and resulting cutting surface characteristics in AWJ have been extensively studied for conventionally fabricated metals [13,14,15]. Janković et al. have developed mathematical models relating AWJ process parameters to surface roughness, based on experimental data [4]. The results confirmed the characteristic categorization of the cutting surface into a smoother upper zone and a sheared lower zone and showed that traverse speed, abrasive mass flow and workpiece thickness are among the main influential factors determining the width of cut, taper of cut, and surface roughness. The authors also pointed out that standards describing the quality of AWJ cut were not yet fully established at the time of publication, which later motivated the development and introduction of quality classifications such as levels Q1–Q5 [4,9,16]. For austenitic stainless steels, Llanto et al. used Taguchi design and ANOVA in AWJ cutting of AISI 304L and showed that increasing water pressure and abrasive mass flow rate improved surface integrity and material removal rate, while increasing traverse speed above the optimum value led to an increase in roughness; material thickness had the highest contribution to the variability of the responses [5]. Löschner et al. focused specifically on AISI 316L steel and investigated the effect of traverse speed on surface quality for 10 mm thickness, confirming significant surface quality degradation at high traverse speeds [6]. Parthiban et al. analyzed AWJ cutting of thin (2 mm) AISI 316L sheets, developed empirical models of the upper and lower kerf widths as a function of abrasive flow, traverse speed, and stand-off distance, and identified parameter combinations that minimized kerf width and improved edge quality using a factorial design [7]. The results of Begic-Hajdarevic et al. confirm the dominant influence of traverse speed and abrasive flow on surface roughness in AWJ machining [8]. When machining AM materials such as 316L using conventional and hybrid chipping operations and special abrasive techniques, Medibew et al. showed that grinding, milling, polishing, lapping, and honing can achieve significant roughness reduction on L-PBF steel and nickel alloys, while sandblasting, abrasive jet machining, and electrochemical methods are particularly promising for complex internal geometries [12]. However, the review also points out that the inherent porosity, staircase effect, and partially melted particles on AM surfaces complicate the action of free or bonded abrasives, which can lead to brittle grain fracture and inhomogeneous removal [12]. Aziz et al. documented how the build orientation, scanning strategy and energy density in AM processes affect not only the microstructure and mechanical properties of 316L, but also the initial surface condition and susceptibility to various post-processing strategies [10]. Specifically, at the interface of AWJ technology and additively manufactured 316L steel, an important contribution is the work of Vandžura et al. [17], which focused on the formation of non-transient erosion grooves during AWJ machining of SLM-produced SS 316L stainless steel. The authors applied the full factorial DoE method, in which they varied the traverse speed, stand-off distance, and abrasive mass flow pressure of 50 MPa and evaluated their effect on the groove shape and the volume of material removed. A key aspect of their approach was the machining of the material in two planes with respect to the layering direction during SLM, which allowed an explicit comparison of the response of the material with different mechanical properties resulting from the additive process. They demonstrated that the additively fabricated SS 316L steel and its layering direction significantly affect the erosion groove shape, material removal efficiency, and sensitivity to changes in process parameters. This means that the erosion mechanisms are closely linked to the orientation of the samples during building [17]. This study focuses on erosion grooves created with low pressure and does not evaluate full-scale parting cuts at industrially used quality grades Q1–Q5. Despite the widespread use of AWJ in parting of conventionally manufactured steels and the growing body of work on abrasive finishing of AM metals, very few studies have addressed AWJ cutting as a post-processing method for additively manufactured 316L in the context of industrially relevant cuts. Existing reviews have mainly presented AWJ as one of the unconventional technologies in the broader context of abrasive machining [10,12], but do not provide systematic data on how AWJ cut quality, roughness, and surface integrity depend on AM-specific factors such as print orientation, layer thickness, etc. There is also a lack of studies that quantitatively link the industrially used AWJ quality classes (Q1–Q5) for AM steels with roughness parameters and microstructural damage according to relevant standards [9,16]. Based on the above, it can be concluded that for conventionally produced alloys, the influence of AWJ parameters on cut quality, cut geometry and surface roughness is relatively well investigated [4,5,6,7,8]. For additively produced 316/316L, there are extensive reviews focused on process parameter optimization, defect mitigation, and post-processing strategies, including a wide range of abrasive finishing processes [10,12]. Moreover, Vandžura et al. clearly showed that the different mechanical properties and anisotropy of SLM 316L due to the layering direction fundamentally affect the material response to AWJ erosion and that the mechanical behavior of additively prepared steel should be explicitly considered when designing AWJ processes. Nevertheless, systematic studies that simultaneously combine the anisotropic nature of SLM 316L (build orientation parallel vs. perpendicular to the cutting plane), and AWJ quality classes Q1–Q5 as relevant process settings are still lacking [17].

The present study is designed to address this research gap, where two sets of samples with different build orientations are produced by AM technology, which are subsequently cut by AWJ with process parameter settings corresponding to quality classes Q1–Q5. The aim of the work is to characterize the influence of AWJ quality class (Q1–Q5) on surface roughness parameters (Ra, Rz) and 3D topology of the SLM 316L cut surface for two mutually orthogonal build orientations. SEM and EDX methods are applied to detect the occurrence of defects and residual abrasive particles (grit embedment) on the cut surfaces.

## 2. Materials and Methods

### 2.1. Motivation, Methodology, and Methods

The presented study is focused on the evaluation of the surface properties of AM- produced SS 316L stainless steel after sectioning by abrasive waterjet (AWJ) technology. The experiment was designed to observe the changes of surface roughness and morphology depending on the set quality levels of the cut (Q1–Q5) and different orientation of the material layering during the SLM process. The experimental methodology was designed to allow for the comparison of samples cut along the direction of the layers and perpendicular to the layers. The motivation of the experiment is to investigate the parameters of the AWJ cut, represented by the quality of the cut surfaces (Q1–Q5) and their dependence on the properties of the AM-produced material.

Quantitative surface evaluation in this study was based on linear roughness parameters Ra and Rz. 3D optical microscopy was used primarily for qualitative visualization of cut-surface morphology.

An experimental methodology combining both macroscopic and microscopic analytical methods is proposed for the evaluation of the AWJ cut surface of the experimental SS 316L material produced by the SLM method. The methodology is designed to systematically investigate the influence of different quality levels of the section (Q1–Q5) on the resulting surface properties. The experimental procedure includes surface topography characterization, surface roughness measurement, SEM analysis of microstructural evaluation, and EDX spectroscopy of chemical composition analysis.

The experimental methodology consists of several main steps: preparation of AM- produced material samples; setting of AWJ process parameters under controlled conditions, optical and scanning electron microscopy (SEM) for surface morphology analysis and detection of material changes, roughness measurements for evaluation of cut quality, and EDX analysis for determination of chemical composition of surface areas; and detection of potential contamination by abrasive particles. The main goal of these methods is to identify the presence of residual abrasive particles, quantify the differences in surface morphology at different cutting process parameters, and evaluate plastic deformations caused by the AWJ process. These analytical approaches allow a detailed understanding of the influence of AWJ process parameters on the final surface condition of AISI 316L. The results of the study will contribute to the optimization of the AWJ cutting process and the exploitation of the potential of this method in cutting additively prepared materials.

### 2.2. Experimental Procedure and Preparation of Experimental Specimens

The Water Jet 3015 RT-3D (Kovostrojservis, spol. s.r.o, Pardubice, Czech Republic, located at Technical university Košice, Faculty of manufacturing technologies, Prešov, Slovakia) system with a working area of 3000 mm × 1500 mm was used for cutting the experimental samples. Pressurized water was generated by Jets PTV 3.8/60 high-pressure pump, which can generate water pressure in the 50 MPa and 380 MPa modes. The nozzle of the AWJ system used has a standard geometry. The diameter of the water nozzle orifice was 0.33 mm, the diameter of the focusing tube was 1.02 mm, and its length was 76.2 mm. The cutting head was set to perpendicular cut (90°). This configuration is optimized for the process parameters for the cutting with the Mesh 80 garnet abrasive.

The water pressure used, 380 MPa, allows for precise adjustment of process parameters, which is crucial for optimizing the cutting process depending on the material properties. Each quality level (Q1–Q5) is determined based on precisely defined criteria of surface topography and cut homogeneity, with the highest quality (Q5) representing a smooth, even surface with minimal roughness, while quality Q1 corresponds to a rougher, less homogeneous surface. To achieve and maintain consistent cut quality requirements, precise process parameters are specified for each quality level, including water pressure, abrasive flow rate, nozzle distance from material, and feed rate.

The experimental samples were produced by SLM technology from powder material Renishaw SS 316L-0407 (Renishaw plc, Wotton-under-Edge, UK). The resulting alloy is an austenitic stainless steel with the main alloying elements of chromium up to 18%, nickel up to 14%, and molybdenum up to 3%. Powder materials from Renishaw are supplied according to strict specifications to minimize batch-to-batch variation [18]. The composition of the powder material is listed in Table 1.

It is important to note that the mechanical properties of AM alloys produced by SLM are influenced by the material preparation process itself. The material has different properties resulting from the layering and material preparation (Table 2).

Material specimens for the experiments were produced on a Renishaw AM500E. (Renishaw plc, Wotton-under-Edge, located at Protolab, Ostrava, Czech Republic) The AM500E system with a working chamber of 250 × 250 × 350 mm^3^ operates with a laser power of up to 500 W, with a maximum scanning speed of 2000 mm·s^−1^, and layer thickness in the range 20 μm to 100 μm. Optimal process parameters were used according to the recommendations of the powder material manufacturer, which guarantee the production of parts with maximum quality and properties similar to conventional AISI 316L. The set printing process parameters: the laser power P for melting the powder was set to 200 W, the scanning speed v 650 mm·s^−1^, and the hatch spacing d was equal to 110 μm. The layer thickness t was 50 μm. The specimens were printed in a protective atmosphere of argon (Ar). The scanning strategy for individual layers defining the path of the laser during building was set to meander.

**Figure 1:**
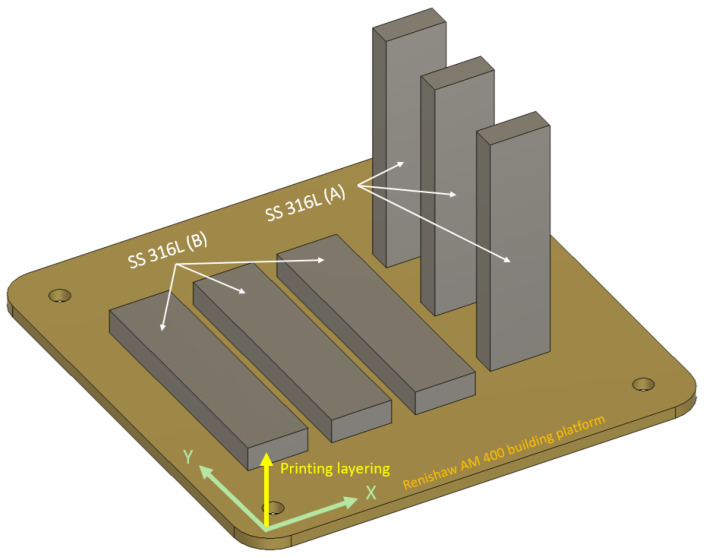
Illustration of the specimens layout on the building platform.

The dimensions and geometry of the specimens were designed with respect to the planned experiments and the different mechanical properties resulting from the print direction. The specimen dimensions (X, Y, Z) were 25 × 100 × 10 mm^3^ and 25 × 10 × 100 mm^3^. According to the print direction (Figure 1), the SS 316L specimens were designated as SS 316L A and SS 316L B.

For SS 316L B specimens, a total of 200 layers were printed in the Z-axis direction, with a layer thickness of 50 μm, which corresponds to a total sample height of 10 mm. For SS 316L A specimens, the specimen height was 100 mm, which at the same layer thickness corresponds to 2000 layers. In this case, the layering direction is oriented longitudinally in the direction of the specimen z axis. For the purposes of the experiments, two series of experimental specimens were produced with respect to the layering of the material during building. The experimental specimens were produced together on one printing substrate using the same process parameters.

**Figure 2:**
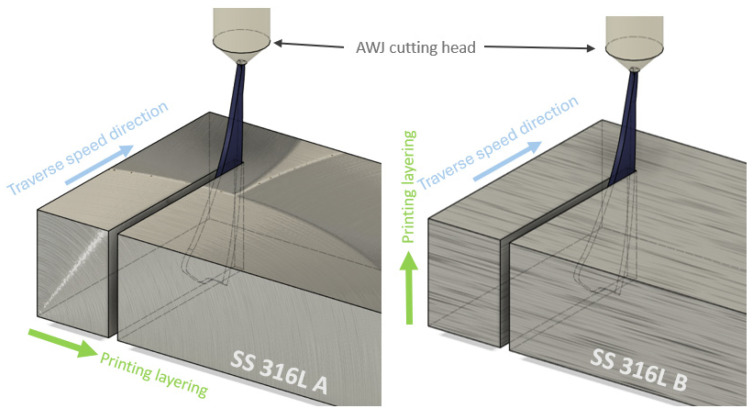
Schematic representation of the AWJ cutting process.

Samples of AM-produced SS 316L material can be more difficult to cut due to their additive manufacturing process and the resulting different material properties with respect to the direction of layering of the material during printing. Therefore, the experiments were set up so that the additive material SS 316L was machined with an abrasive waterjet perpendicular to the layering direction (SS 316L B) as well as parallel to the layering direction (SS 316L A). The prepared material samples were cleaned and dried before the cutting process to remove all impurities that could affect the quality of the cut. The process is shown in Figure 2.

The technologic parameters of the cutting (Table 3) were set in the Igems control program according to the machinability of the conventional stainless steel. The main technological parameters for individual cut qualities were Q1–Q5. The technological parameters settings for individual qualities were chosen so that the variable parameter was the traverse speed v_p_ (mm·min^−1^).

**Figure 3:**
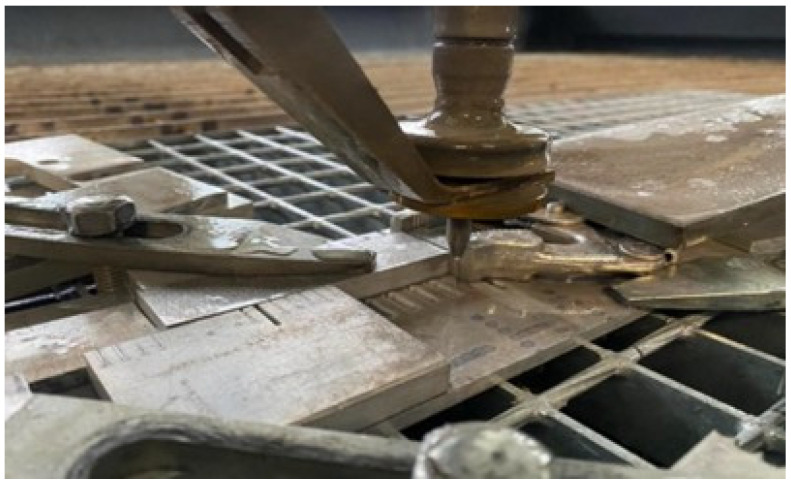
Experimental cutting of AISI 316L specimens using AWJ technology.

The cutting process was carried out for three sets of dimensionally identical specimens of materials SS 316L A, SS 316L B). The experimental process is shown in Figure 3.

## 3. Results

### 3.1. Evaluation of Topography and Surface Roughness Parameters

**Figure 4:**
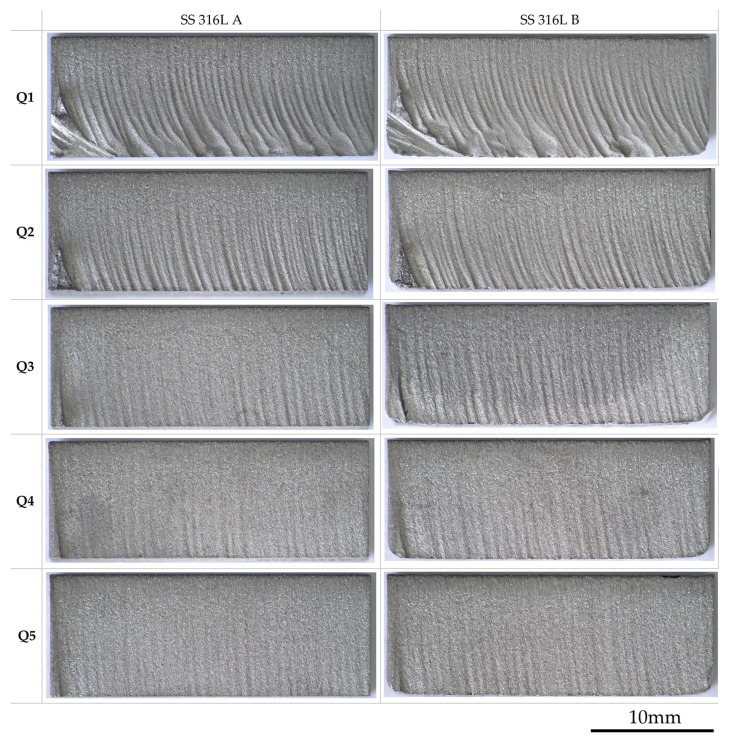
Surface topography of specimens SS 316L A/B.

The topography of the cut surfaces of experimental materials with the respective qualities Q1–Q5 shows significant differences in dependence on parameters of the AWJ process. In the presented images of cut qualities (Figure 4) can be observed the characteristic features of individual cut qualities, while the jet lag across the cut qualities (Q1–Q5) is clearly visible. With increasing traverse speed of the cutting head at the same mass flow of abrasive, the gradual kinetic energy loss of the abrasive waterjet leads to an increased occurrence of surface defects and deterioration of the cut quality. This effect is especially pronounced at lower quality cut levels (Q1, Q2), where arcuate grooves and irregular grooving can be observed, especially in the lower zone of the samples. Jet lag in these cases leads to incomplete separation of the material, which required subsequent mechanical separation of these parts. Comparing the samples SS 316L A and SS 316L B, it can be observed that this effect is more pronounced in SS 316L B, which indicates a higher susceptibility of this layering orientation to unstable erosion mechanisms. When moving to higher quality levels (Q3–Q5), a visible smoothing of the surface occurs, while at the best cut quality (Q5) the surface is the most homogeneous with minimal traces of abrasive action. This effect is caused by the lower traverse speed of the cutting head, which allows a longer effect of the abrasive jet on the material, resulting in more effective removal without the formation of secondary defects. In the comparison between SS 316L A and SS 316L B, it is observed that orientation B tends to show a slightly rougher structure even at higher quality levels, which confirms the influence of the directional layering of the material on the cutting process.

**Figure 5:**
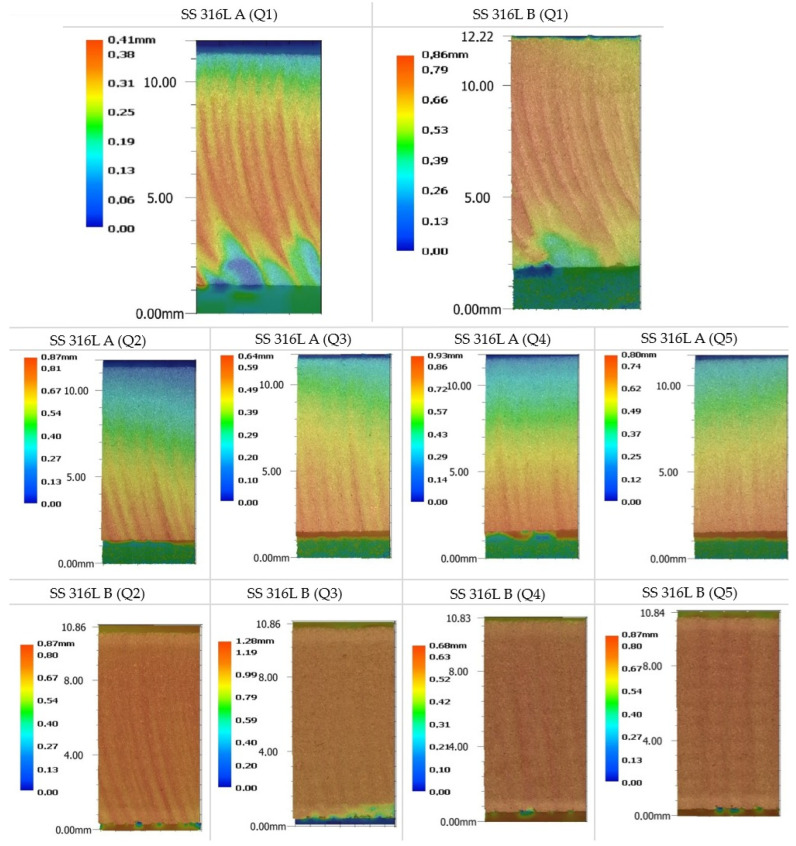
3D topographies of sample surfaces obtained with a Keyence VHX-7000 microscope.

Evaluation of the roughness of machined surfaces of additive stainless steel by abrasive waterjet (AWJ) technology is crucial for understanding the influence of process parameters on the resulting functionality and integrity of the material. Measurement and analysis of surface roughness parameters provide important data that allow optimizing technological parameters and preventing defects. The cut surfaces were scanned and analyzed using the optical macroscopy technique, which allows for a detailed assessment of the surface topography and surface homogeneity. This method provides fast and accurate data on the macroscopic indicators of the surface, including the identification of irregularities, roughness, and possible defects. A 4K 3D optical microscope Keyence VHX 7000 (KEYENCE Corporation, Osaka, Japan, located at Technical university Košice, Faculty of manufacturing technologies, Prešov, Slovakia) was used to scan and evaluate the surfaces, which, thanks to its high resolution and the ability to scan the surface in three dimensions, enables comprehensive analysis. The microscope allows for a detailed 3D reconstruction of the surface, thus enabling accurate measurement of height differences, grooves and elevations. Using a 4K 3D optical microscope, it was possible not only to evaluate the surface but also to identify surface defects, and also to quantify the differences between individual qualitative levels of sections (Q1–Q5), thus ensuring an objective quality analysis. Scanning of individual sample surfaces was performed with the same device settings for all evaluated sample surfaces. Individual 3D scans and topographies of the scanned surfaces are shown in Figure 5.

**Figure 6:**
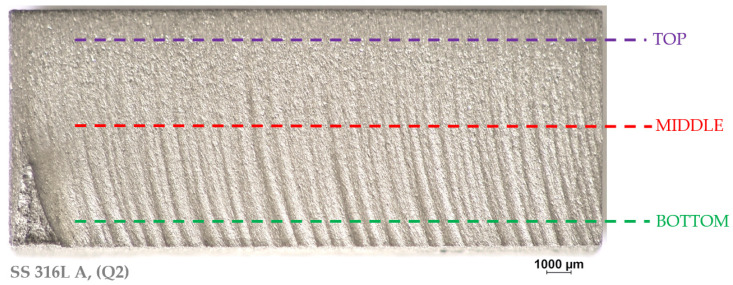
Locations of roughness parameters evaluation on AM samples.

The surface roughness assessment locations were selected to provide a comprehensive view of the cut quality throughout its entire course. An example representation of the roughness assessment location is shown in Figure 6.

**Figure 7:**
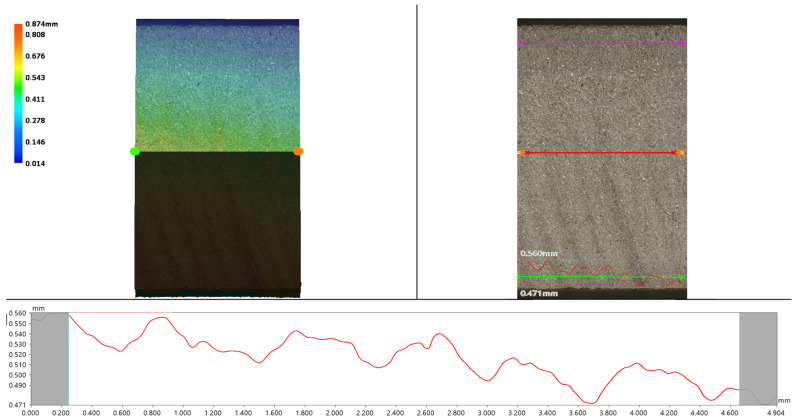
Visualization of measurement 316L A, Q2.

BOTTOM (1 mm from the lower edge) is a key location where the bottom of the cut is most affected by the abrasive reflection and the waterjet exit, often resulting in higher roughness. This area has a major impact on the functional properties of the part. MIDDLE (5 mm from the bottom edge) represents the stable part of the cut and TOP (1 mm from the upper edge) is the area closest to the jet entry into the material. This area usually has lower surface roughness, which allows for analysis of the efficiency of the cut initiation. The aim of these measurements was to identify how the surface roughness values change depending on the cut quality (Q1–Q5), the measurement height, and especially the layering direction of the additively produced material. Data acquisition from the roughness evaluation with the Keyence VHX 7000 optical microscope is shown in Figure 7.

**Figure 8:**
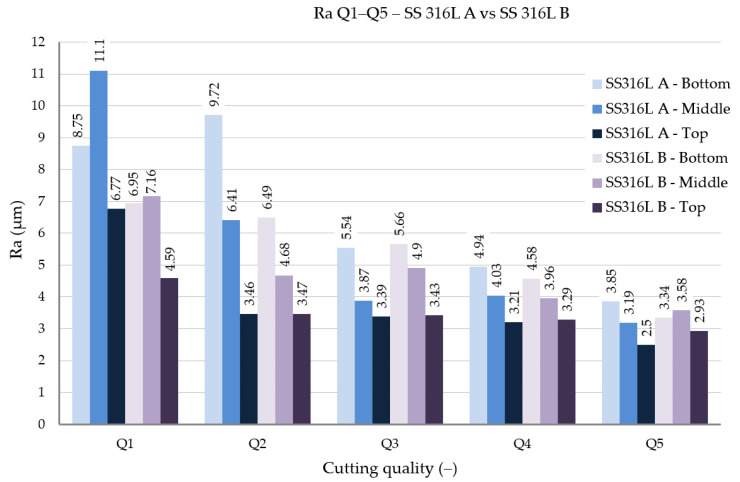
Roughness parameter Ra Q1–Q5, SS 316L A vs. SS 316L B.

**Figure 9:**
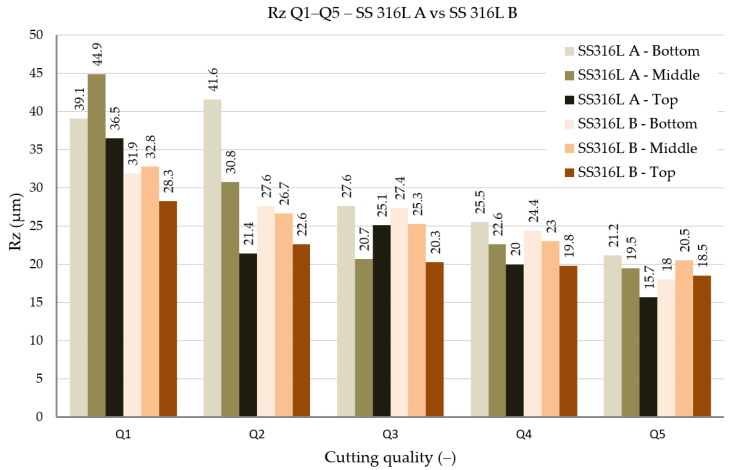
Roughness parameter Rz Q1–Q5, SS 316L A vs. SS 316L B.

The roughness parameters Ra (arithmetic mean deviation of the profile) and Rz (maximum height of the profile) were evaluated. The results of individual measurements are shown in Figure 8 and Figure 9.

The experimentally obtained results confirmed that the lowest Ra values were generally achieved at the TOP evaluation line for the best cut quality (Q5). At this position, SS 316L A reached Ra = 2.5 μm, while SS 316L B reached Ra = 2.93 μm. A similar trend was observed toward lower cut quality (Q1), where Ra increased to 8.75 μm for SS 316L A and 6.95 μm for SS 316L B. However, this does not imply that SS 316L B generally exhibited lower roughness. At the MIDDLE evaluation line for Q1, SS 316L A showed a higher mean Ra value (11.1 μm) than SS 316L B (7.16 μm), indicating fluctuation in roughness caused by the uneven distribution of erosion forces in the interlayer areas.

SS 316L A showed a more consistent roughness profile across all cut heights, confirming the more stable resistance of the layered structure to longitudinal loading by an abrasive jet.

In terms of the Rz parameter, which takes into account the maximum profile height and thus more significantly describes the presence of local defects or deep grooves, a similar trend was confirmed. The lowest Rz values were again observed for quality Q5 in the TOP position, where it reached 15.7 μm for SS 316L A materials, while SS 316L B had 18.5 μm. In the BOTTOM position, values of up to 21.2 μm (A) and 18.0 μm (B) were achieved for the same quality. However, for the worst quality Q1, the difference was already more significant, with SS 316L A reaching an Rz of up to 44.9 μm at the MIDDLE evaluation point compared to 32.8 μm for SS 316L B. The higher Rz value in this case may be related to the trapping of abrasive particles and their random arrangement in the layer structure of SS 316L A, which cause transient jumps in the surface profile.

Two build orientations were compared: SS 316L A, where the material layers were aligned with the cutting direction, and SS 316L B, where the cut was made perpendicular to the layer orientation. These orientations are important from a materials perspective because SLM-based additive manufacturing introduces anisotropy in mechanical properties due to directional solidification and layer-wise material deposition. SS 316L A generally exhibits higher ductility and better plastic deformation behavior along the layering direction, which is reflected in higher resistance to microcracking and surface damage during AWJ cutting. In contrast, SS 316L B, cut perpendicular to the layers, is more susceptible to delamination, microcrack formation, and abrasive particle entrapment at interlayer boundaries, which can increase the resulting roughness.

The analysis showed that layer orientation has a fundamental influence on the post-AWJ surface morphology. SS 316L A, where the jet direction was parallel to the layers, exhibited a more homogeneous roughness distribution across cut depth and lower Ra and Rz variability at quality levels Q3–Q5. In contrast, SS 316L B was more sensitive to cut-quality changes and generally showed larger deviations between TOP and BOTTOM regions, indicating a dynamic interaction between the jet and the oriented layer microstructure. These findings highlight the importance of part orientation during additive manufacturing, particularly when subsequent abrasive waterjet cutting is planned. From this perspective, post-AWJ surface roughness is not only a function of process parameter settings, but is also strongly influenced by the internal microstructure and directional anisotropy of the material. Overall, the effect of build orientation on roughness was position-dependent; although orientation B showed more frequent surface defects in SEM observations, Ra/Rz values were not uniformly higher for B at all positions and quality levels.

### 3.2. SEM Analysis of Surface

Scanning electron microscopy (SEM), which enables high-resolution imaging of surface structures of materials with high depth of field, was used for detailed analysis of the cut surface of specimens. In this study, the Tescan MIRA3 (TESCAN, Brno, Czech Republic, located at Slovak academy of sciences, Košice, Slovakia) device was used, which works on the principle of interaction of accelerated electrons with the surface of the samples, which allows detailed mapping of the microstructure and detection of local defects caused by abrasive waterjet cutting. Before the SEM analysis itself, the samples were carefully prepared to minimize unwanted effects on the obtained results. The preparation process included ultrasonic cleaning of the surface to remove loose abrasive particles and drying of the samples, ensuring their optimal observation in the electron microscope. SEM images were acquired at different magnifications (25×, 100×, 500× and 3000×), focusing on assessing the surface topography, identifying areas with different levels of erosion and the presence of secondary defects such as microcracks or abrasive residues. The selection of the correct electron beam accelerating voltage was crucial for achieving optimal contrast and detail resolution. In this case, an accelerating voltage of 20 kV was used, which provides a balanced compromise between electron penetration depth and resolution of surface details.

**Figure 10:**
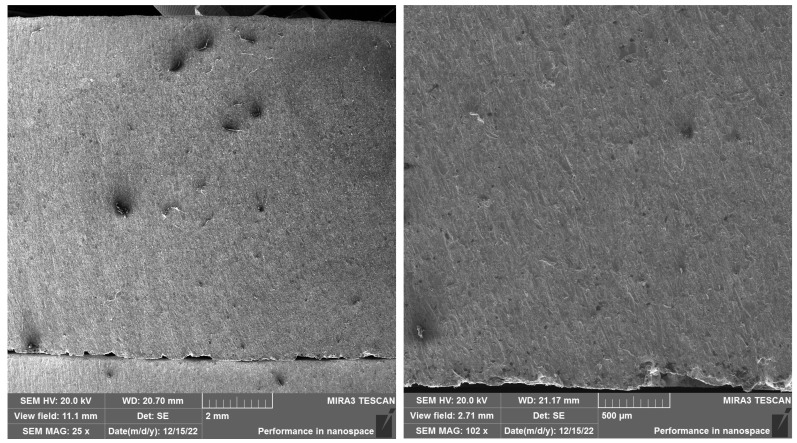
SEM SS 316L B magnification left 25×, right 100×.

In the study, SEM analyses were focused on samples cut with the best cut quality (Q5). This selection was made intentionally because at this quality the process parameters are most optimized and the lowest level of surface defects is assumed, which allows a thorough comparison of the influence of the layering direction on the resulting section. The evaluation provides a comprehensive picture of how different layer orientations (cut in the direction of the layers—SS 316L A, and cut perpendicular to the layers—SS 316L B) are reflected in the cut quality under otherwise identical conditions. When comparing the surface topography of the SS 316L material (A), (B), we observe different surface topography caused by sectioning this material in different directions. In the SEM images (Figure 10) of SS 316L B sectioned with the Q5 quality, we can observe defects, impurities, and abrasive particles at 25× and 100× magnifications.

**Figure 11:**
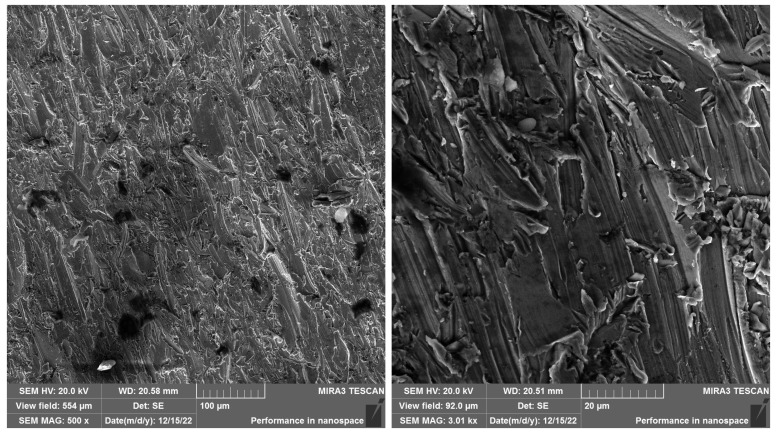
SEM SS 316L B magnification left 500×, right 3000×.

At 500× magnification of specimen 15, it can be observed how the technological parameters of the cut quality Q5 have disrupted the interparticle connection. Figure 11 shows fragmented particle of the embedded abrasive. At 3000× magnification, cleavage of facets is evident on the surface of the observed area.

**Figure 12:**
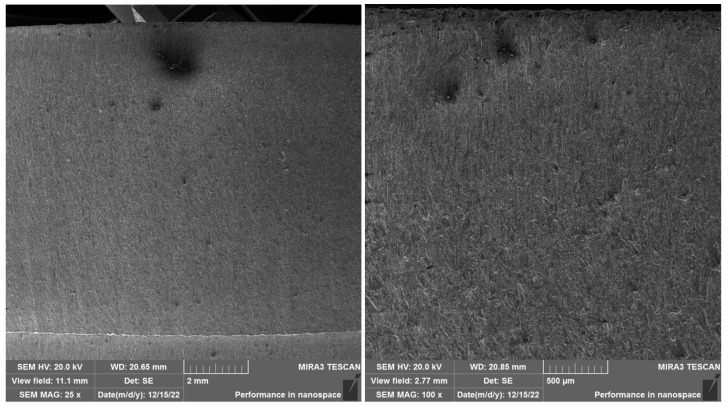
SEM SS 316L A magnification left 25×, right 100×.

**Figure 13:**
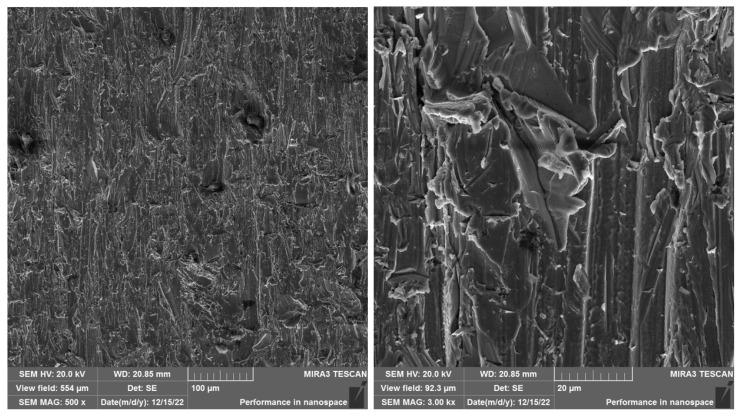
SEM SS 316L A magnification left 500×, right 3000×. When comparing the SS 316L A and SS 316L B samples cut with Q5 quality (best cut quality), there are clear differences in the surface topography depending on the direction of the material layering. In the case of the SS 316L A sample (cut in the direction of the layers), the surface is homogeneous at high magnification, without obvious occurrence of embedded abrasive particles, with a fine granular structure without signs of disruption of interparticle bonds. The locations for analysis were selected randomly, which may be one of the reasons for the absence of abrasive residues in the evaluated area. On the contrary, in the case of the SS 316L B samples (cut perpendicular to the layers), abrasive particle residues, microcracks, and disruption of the surface structure were recorded already at lower magnifications, which indicates a higher susceptibility of this orientation to erosion damage. Fragmented particles and structural defects were also present at high magnification (3000×), which may be related to the disruption of the bonds between individual sintered layers when they are loaded perpendicularly by the AWJ current. These differences confirm the importance of the layering direction in evaluating the processability of additively manufactured materials using the AWJ technology. Orientation A demonstrated greater resistance to microscopic damage and lower levels of abrasive contamination, confirming the suitability of slower traverse speed and optimized cutting for precise parting.

In SEM images of SS 316L A specimens (Figure 12 and Figure 13) cut with the best quality, Q5, can be observed a significantly more uniform and smoother surface topography compared to lower cut qualities. At lower magnifications (25× and 100×), the surface is compact, without visible grooves, microscopic cracks, or significant morphological defects. No embedded abrasive particles were observed in the locations selected for analysis, which can be attributed both to the slower AWJ traverse speed and to the random selection of the evaluated areas, which may not have contained areas with a higher probability of abrasive capture.

At higher magnifications (500× and 3000×), the surface is characterized by a fine granular structure, without disturbed interparticle bonding. The absence of secondary defects confirms the beneficial effect of optimized process parameters for the Q5 quality, where the highest quality is achieved without significant surface damage.

### 3.3. EDX Analysis

**Figure 14:**
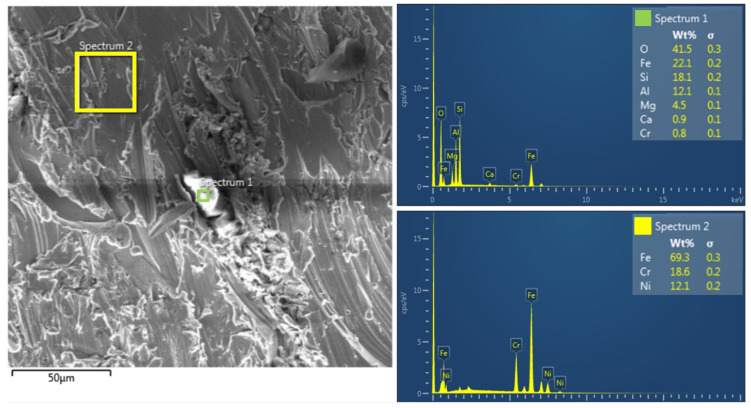
EDX analysis of specimen SS 316L A quality Q5 (highest).

**Figure 15:**
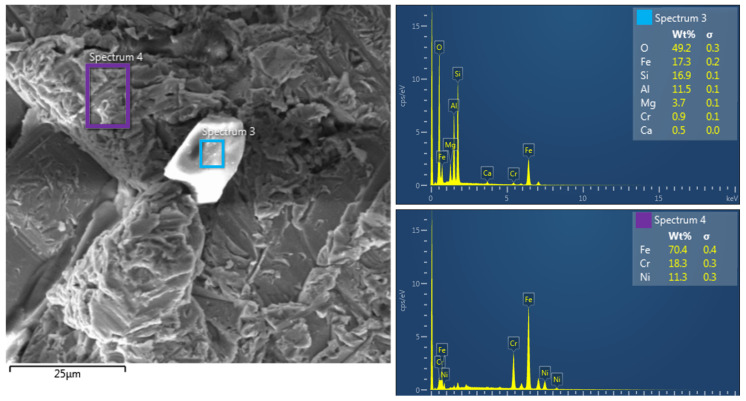
EDX analysis of specimen SS 316L A quality Q1 (lowest).

Energy Dispersive X-ray Spectroscopy (EDX) was also used to complement the SEM analysis, which allows for the quantitative and qualitative determination of the chemical composition of the investigated areas. This technique uses X-rays emitted during the interaction of electrons with the sample, thereby obtaining spectral information about the elemental composition of the surface. EDX analysis was performed on the specimens in the central area 5 mm from the bottom edge where the abrasive residues are most likely to occur, to confirm the presence of elements originating from the abrasive used (Australian garnet mesh 80), mainly silicon (Si), aluminum (Al), and oxygen (O), which are characteristic of the garnet abrasive used, and whether the AWJ process affected local contamination with abrasive particles. In addition, areas of the base material were also analyzed to confirm the composition of the material, whether the AWJ process affected its chemical composition or whether local contamination with abrasive particles occurred. Figure 14 and Figure 15 show images of samples of the experimental material SS 316L A, with the worst Q1 and best Q5 cut quality.

EDX analysis of Spectrum 1 and Spectrum 3 areas showed high contents of oxygen (O), silicon (Si), aluminum (Al), and magnesium (Mg), indicating the presence of garnet abrasive, which was used in the abrasive waterjet cutting (AWJ) process. The presence of these elements indicates that in the investigated areas, abrasive particles were trapped in microgrooves and surface irregularities of the cut specimen. In addition, a low concentration of iron (Fe) was detected, confirming that the analyzed areas do not represent the primary material of the specimen, but rather abrasive residues adhered to the surface. EDX spectral analysis of Spectrum 2 and Spectrum 4 areas revealed a chemical composition of elements typical of 316L stainless steel, with the dominant elements being iron (Fe), chromium (Cr), and nickel (Ni). The absence of silicon (Si) and aluminum (Al) elements in these spectra confirms that these are areas without residual abrasive particles, indicating more efficient removal or lower contamination levels compared to Spectrum 1 and 3. The higher concentration of chromium (Cr) and nickel (Ni) is consistent with the chemical composition of 316L stainless steel, while a moderate concentration of oxygen (O) was also detected, which may indicate possible surface oxidation due to the mechanical and chemical action of the waterjet with the abrasive.

## 4. Discussion

The results of this study showed that the surface quality after the cutting of additively manufactured SS 316L steel by AWJ technology is determined by a combination of process parameters and the direction of material layering during SLM. The roughness values Ra and Rz showed a consistent trend depending on the quality class of the cut (Q1–Q5).

The lowest values were achieved at the best cut quality (Q5), with the Ra parameter at the top of the cut reaching 2.5 μm (SS 316L A) and 2.93 μm (SS 316L B). The Rz parameter at the same position was 15.7 μm (A) and 18.5 μm (B). These results confirm that the lower traverse speed of the cutting head allows for a more efficient action of the abrasive waterjet and leads to a more homogeneous surface topography at the entrance to the material.

At the worst cut quality (Q1), Ra values of 8.75 μm (A) and 6.95 μm (B) were measured. The differences were more pronounced for the Rz parameter, where in the middle of the cut (MIDDLE), SS 316L A reached up to 44.9 μm compared to 32.8 μm for SS 316L B. This result indicates that under deteriorated cutting conditions, the anisotropic structure is more sensitive to the direction of AWJ action, while the cut perpendicular to the layers (B) may locally show lower roughness, but with a higher defect rate.

Studies focused on machining stainless steels consistently confirm the characteristic cut surface gradient, with the lowest roughness at the jet entry and a progressive deterioration toward the jet exit, mainly caused by traverse speed and the loss of abrasive water- jet energy with increasing depth. Llanto et al. [5] showed that a lower traverse speed improves surface quality (AISI 304L); however, differences in the absolute values of the evaluated parameters cannot be directly compared with our AM 316L due to differences in the material and in the process parameter settings. Löschner et al. [6] reported for AISI 316L with a thickness of 10 mm, at the excellent quality grade, a value of Ra ≈ 2.69 μm in the upper region (IN), which increased toward the lower region (OUT) to Ra ≈ 5.16 μm, while Rz increased from ≈17.53 μm (IN) up to ≈27.44 μm (OUT). This is quantitatively consistent with our values for quality Q5 in the TOP region (Ra = 2.5 μm, Rz = 15.7 for SS 316L A; Ra = 2.93 μm, Rz = 18.5 μm for SS 316L B). Perec et al. [20] studied Sq roughness on the upper cut area on 1.4923/X22CrMoV12-1 stainless steel. Results showed variation in Sq values in the range 2.5 to 6 μm in accordance with changing process parameters. Szatkiewicz et al. [21] studied additively manufactured composite S316L/polymer after AWJ cutting. The study highlighted Sq roughness 5.6 μm on the A316L component and a concurrent Sq not exceeding 1.26 μm on polymer component. Żyłka, Łet al. [22] concluded that traverse speed exhibits greater impact on the Rt roughness parameter compared to the Ra parameter. The study [23] observed roughness parameter Ra in three zones of cut area (the up zone, middle zone, and down zone). Authors highlighted the values of Ra 2.597 μm in the up zone, 3.006 μm in the middle zone, and 4.105 μm in the down zone.

SEM analyses provided a more detailed view of the surface morphology. SS 316L A samples cut with quality Q5 had a homogeneous surface without embedded abrasive particles, while SS 316L B samples showed microcracks and fragments of garnet abrasive already at a magnification of 500×. EDX analysis confirmed these observations by detecting Si, Al, and O elements in the surface regions, indicating the presence of residual abrasive particles.

These findings are consistent with the knowledge of the anisotropy of mechanical properties of additively manufactured metals. The orientation of the layers plays a significant role in the interaction with the AWJ—a longitudinal cut (A) provides a more stable roughness profile, while a cut perpendicular to the layers (B) is more susceptible to erosion damage and surface contamination.

## 5. Conclusions

Based on the results obtained from the experiments, the following conclusions can be drawn:

- The set process parameters for the AWJ cut have a direct impact on the surface quality. The main variable parameter was the traverse speed v_p_ depending on the selected quality. The other technologic parameters remained firmly defined (abrasive mass flow m_a_, stand-off distance SoD, and water pressure p). The lowest roughness values are achieved for quality Q5 (Ra ≈ 2.5–2.9 μm; Rz ≈ 15.7–18.5 μm), while for quality Q1 the values are significantly higher (Ra up to 8.75 μm; Rz up to 44.9 μm).
- The orientation of the layering during the preparation of the experimental material by the SLM method affects the resulting surface morphology of the individual cuts. The material designated as SS 316L A (cut in the direction of the layers) shows a more homogeneous topography and lower roughness variability, while the experimental material designated as SS 316L B (cut perpendicular to the layers) is more prone to microcracks and abrasive particle trapping. SEM and EDX analyses confirmed the presence of abrasive fragments, which documents the sensitivity of the material to contamination by the used abrasive.

The practical benefit of the study lies in the application of the AWJ technology to the cutting of additively manufactured components, primarily due to the advantages that this technology brings. The most important benefit is the absence of a heat-affected zone (HAZ), regardless of their hardness or thermal conductivity. The AWJ method allows not only precise cutting, but also effective removal of support structures in the context of additive manufacturing. The disadvantage of AWJ technology is the possible contamination of the surface with residual abrasive particles, which, as confirmed by the results of EDX analyses, can be trapped in microgrooves and interlayer areas, especially when cutting perpendicular to the layering direction. Such contamination can adversely affect the surface integrity of the part and its subsequent processing. Another risk is surface corrosion, which can be accelerated by the presence of abrasive particles. Another disadvantage is lower productivity when a high cut quality is required (Q5), where it is necessary to choose low traverse speed.

From the perspective of the study results, it can be concluded that for applications requiring high surface quality, it is more advantageous to orient the cut in the direction of the layers (SS 316L A) and choose lower feed rates, i.e., a higher cut quality. In this way, surface defects and contamination can be minimized, which increases the applicability of AWJ technology in the processing of additively manufactured components.

## Figures and Tables

**Table 1:** Composition of Renishaw SS 316L powder material [18].

Element	Fe	Cr	Ni	Mo	Mn	Si	N	O	P	C	S
Mass (%)	balance	16–18	10–14	2–3	≤2	≤1	≤0.10	≤0.1	≤0.05	≤0.03	≤0.03

**Table 2:** Mechanical properties of Renishaw SS 316L [19].

Property	As Built
Bulk density	≥99.8%
Ultimate tensile strength (XY) ^1^	693 MPa ± 3.5 MPa
Ultimate tensile strength (Z) ^2^	631 MPa ± 3 MPa
Yield strength (XY) ^1^	565 MPa ± 3.5 MPa
Yield strength (Z) ^2^	495 MPa ± 3 MPa
Elongation after fracture (XY) ^1^	53% ± 2%
Elongation after fracture (Z) ^2^	47% ± 1%
Modulus of elasticity (XY) ^1^	205 GPa ± 12 GPa
Modulus of elasticity (Z) ^2^	208 GPa ± 19 GPa
Hardness Vickers, (XY) ^1^	199 HV0.5 ± 3 HV0.5
Hardness Vickers, (Z) ^2^	213 HV0.5 ± 5 HV0.5
Surface roughness Ra, (Z) ^2^	11 µm ± 2 µm
Surface roughness Rz, (Z) ^2^	77 µm ± 14 µm

^1^ (XY)-horizontal. ^2^ (Z)-vertical.

**Table 3:** Technological parameters of AWJ according to Q1–Q5.

Quality Q [−]	Pressure p [MPa]	Stand-Off Distance SoD [mm]	Abrasive Mass Flow m_a_ [g·min^−1^]	Traverse Speed v_p_ [mm·min^−1^]
Q1	380	4	400	394.53
Q2	380	4	400	261.78
Q3	380	4	400	164.22
Q4	380	4	400	117.97
Q5	380	4	400	91.27

## Data Availability

The original contributions presented in this study are included in the article. Further inquiries can be directed to the corresponding author.
